# Major and Minor Group Rhinoviruses Elicit Differential Signaling and Cytokine Responses as a Function of Receptor-Mediated Signal Transduction

**DOI:** 10.1371/journal.pone.0093897

**Published:** 2014-04-15

**Authors:** Bryce A. Schuler, Michael T. Schreiber, LuYuan Li, Michal Mokry, Megan L. Kingdon, Dana N. Raugi, Cosonya Smith, Chelsea Hameister, Vincent R. Racaniello, David J. Hall

**Affiliations:** 1 Department of Chemistry, Lawrence University, Appleton, Wisconsin, United States of America; 2 Department of Microbiology & Immunology, Columbia University College of Physicians and Surgeons, New York, New York, United States of America; 3 Division of Pediatrics, Wilhelmina Children’s Hospital, University Medical Center Utrecht, Utrecht, The Netherlands; Fondazione IRCCS Ca’ Granda Ospedale Maggiore Policlinico, Università degli Studi di Milano, Italy

## Abstract

Major- and minor-group human rhinoviruses (HRV) enter their host by binding to the cell surface molecules ICAM-1 and LDL-R, respectively, which are present on both macrophages and epithelial cells. Although epithelial cells are the primary site of productive HRV infection, previous studies have implicated macrophages in establishing the cytokine dysregulation that occurs during rhinovirus-induced asthma exacerbations. Analysis of the transcriptome of primary human macrophages exposed to major- and minor-group HRV demonstrated differential gene expression. Alterations in gene expression were traced to differential mitochondrial activity and signaling pathway activation between two rhinovirus serotypes, HRV16 (major-group) and HRV1A (minor-group), upon initial HRV binding. Variances in phosphorylation of kinases (p38, JNK, ERK5) and transcription factors (ATF-2, CREB, CEBP-alpha) were observed between the major- and minor-group HRV treatments. Differential activation of signaling pathways led to changes in the production of the asthma-relevant cytokines CCL20, CCL2, and IL-10. This is the first report of genetically similar viruses eliciting dissimilar cytokine release, transcription factor phosphorylation, and MAPK activation from macrophages, suggesting that receptor use is a mechanism for establishing the inflammatory microenvironment in the human airway upon exposure to rhinovirus.

## Introduction

Human rhinovirus (HRV) is the etiologic agent responsible for most common cold infections and the majority of asthma exacerbations, in both children [1] and adults [2], [3]. HRV serotypes are divided into three clades, known as HRV-A, HRV-B, and HRV-C, based upon genetic similarity [4]–[6]. Picornaviruses, including HRV, have an icosahedral capsid measuring approximately 30 nm in diameter and a positive-sense RNA genome of 7.2 kilobases, but they vary in their utilization of host cell-surface receptors to gain entry to cells. Among the HRV-A and HRV-B viruses, major-group HRV binds the intercellular adhesion molecule 1 (ICAM-1) receptor [7], whereas minor-group HRV binds the low-density lipoprotein receptor (LDL-R) [8], [9]. HRV-C viruses bind an as yet unknown receptor [10].

Literature is conflicting as to whether there are strain differences in response to HRV infection. Most literature discussing the kinetics of HRV infection paints a generalized picture of the virus-induced cellular response that is largely focused on the effects of viral replication and does not take into account receptor-mediated signaling [11]–[14]. However, recent studies demonstrate both *in vitro* and *in vivo* strain differences are important in HRV pathogenesis. A study by Rajan et al. using a co-culture system with epithelial cells and peripheral blood mononuclear cells showed that differences in the rhinovirus strain and the host PBMCs used both contribute to changes in the expression of cytokines and chemokines and thus may explain differences in disease [15]. Clinical observations have also suggested that disease severity is associated with strain differences. For example, Denlinger et al. have shown that minor-group HRVs are responsible for higher rates of asthma exacerbation [16] and difference in severity of disease is apparent between clades [17]–[19].

Clearly there are several factors that are key in determining the outcome of HRV infections. Generally speaking, the role of attachment in viral pathogenesis is understudied, particularly within the picornavirus literature. Indeed, most studies of viral pathogenesis focus on the influence of viral proteins translated after the virus has entered the host cell. Because HRV is capable of binding at least three different cellular receptors, this virus provides a unique opportunity to examine the hypothesis that signals initiated through virus binding may play an important role in viral pathogenesis.

Although the differences in receptor utilization have long been appreciated, the early signaling events associated with HRV receptor-mediated signal transduction remain poorly understood. Because HRV binds to a variety of receptors, the activation kinetics of key signaling proteins upon the binding of virus to these different receptors may be important in the cellular inflammatory and antiviral response. Among these signaling proteins, the components of the mitogen activated protein kinase (MAPK) pathway are particularly relevant. MAPK signaling is involved in diverse processes ranging from proliferation to differentiation to, most relevantly, stress responses [20]. The p38 MAPK pathway and the stress activated protein kinase (SAPK)/c-N-terminal kinase (JNK) pathway both respond to stress stimuli (e.g. cytokines), and these pathways have been associated with the production of the inflammatory cytokines chemokine (C-C motif) ligand 2 (CCL2, also known as macrophage chemotactic protein 1/MCP-1) [13], [21] and chemokine (C-C motif) ligand 20 (CCL20, or macrophage inflammatory protein 3 alpha/MIP3-alpha), respectively [22]. Extracellular signal-regulated kinase 5 (ERK5) is another MAPK involved in inducing the production and activation of transcription factors that can regulate the proliferation and differentiation of the cell [23]. Little is known about ERK5 involvement during viral infection [24], [25] or its activation in macrophages [26], [27], although there is substantial literature on ERK5 activation in endothelial cells and neurons [27]. Putative transcriptional regulators of inflammatory cytokines associated with the activation of these MAPKs include activating transcription factor 2 (ATF-2), cyclic AMP response element binding protein (CREB) and CCAAT-enhancer binding protein alpha (C/EBP-alpha). These transcription factors regulate expression of several inflammatory cytokines known to be important in virally induced asthma exacerbations.

Within the lower airway, HRV has opportunity to come in contact with epithelial cells and alveolar macrophages, the predominant immune cells present in the lung. Both of these cell types possess receptors for HRV, both are capable of pro-inflammatory signaling, and each can influence how the other responds to HRV infection [15], [28]. However, macrophages are not a site of productive HRV replication [29], [30]; despite being non-permissive, macrophages secrete many different immune products in response to HRV, which notably include a wide range of cytokines that are capable of both pro- and anti-inflammatory signaling. Therefore, macrophages are important in establishing an inflammatory microenvironment in the lung [31] and can play a role in the immune response to HRV. Indeed, previous studies link HRV receptor-mediated signal transduction in macrophages to a number of biological endpoints associated with inflammation, including activation of inflammation-associated transcription factors such as the nuclear factor κB (NF- κB) [30], [32], release of inflammatory cytokines [13], [30], [33], and the dampening of the macrophage response to bacteria [34].

Our findings demonstrate for the first time that exposure of primary monocytic cells to two genetically similar serotypes of HRV, one major- and one minor- group, elicits differential activation of signaling molecules and transcription factors. This affects the expression of inflammatory mediators CCL2, CCL20, and IL-10, which are important in the establishment of an inflammatory microenvironment. Furthermore, macrophages derived from the leukemic THP-1 cell line showed limited replication of HRV16 but not HRV1A, whereas primary macrophages were non-permissive for either virus. Taken together, our data suggest a mechanism by which the host response to HRV is partially dictated by the signal transduction cascades initiated upon virus ligation to a particular receptor despite the fact that there is no productive viral replication in the human primary macrophage.

## Materials and Methods

### Ethics Statement

The Lawrence University Institutional Review Board approved the protocol used for collecting blood samples from healthy human donors, and all donors provided written informed consent.

### Isolation and Purification/Maturation of Human Blood Monocyte-lineage Cells

Human blood samples were collected from healthy individuals as described previously [13]. Briefly, whole blood was diluted with HBSS (Cellgro, Mediatech, Manassas, VA) and separated by density gradient through Lymphocyte Separation Medium (Cellgro). Leukocytes were collected from the buffy coat interface between the plasma and erythrocyte layers, and remaining erythrocytes were lysed by ACK Lysing Buffer (BioWhittaker, Walkersville, MD). Collected cells were distributed to 12-well tissue culture plates at 1×10^6^ cells per well and cultured in RPMI 1640 (Cellgro) containing 1% penicillin and streptomycin (Invitrogen, Life Technologies, Carlsbad, CA) and 5% sterile-filtered, heat-inactivated human (type AB) serum (BioWhittaker). Monocytes were matured by plastic adherence for 7–10 days until a macrophage phenotype was achieved, as confirmed by flow cytometry. Purified human monocyte-derived macrophages (MDMs) were lifted off the plate with Cell Dissolution Solution (Sigma, St. Louis, MO), and the cell population was evaluated for CD14, CD86 positive cells using antibodies purchased from Becton Dickinson (San Jose, CA) and viability using annexin V (Sigma Chemical Company, St. Louis, MO) by flow cytometry. Cell populations were typically 95% viable and 95% CD14-positive.

### Cell Culture

Human peripheral blood monocytes were cultured in RPMI 1640 (Cellgro) with 5% human AB serum (Cellgro) and 1% penicillin/streptomycin (Gibco, Life Technologies, Carlsbad, CA) at 37°C in a humidified incubator with 5% CO_2_. THP-1 monocytes, obtained from American Type Culture Collection (Manassas, VA) were cultured in RPMI 1640 with 5% fetal calf serum (HyClone**)** and 1% penicillin/streptomycin.

### Preparation of HRV Stocks

HRV serotypes 16 and 1A were gifts from the Jim Gern laboratory at the University of Wisconsin at Madison, and serotypes 2 and 39 were kindly provided by the Vincent Racaniello laboratory at Columbia University. All serotypes are from the HRV-A group. HRV was grown in HeLa cells and subsequently sedimented through a sucrose step gradient to remove exogenous protein and other contaminants. The titer of HRV was determined and the virus stored at −80°C as previously described [35]. RPMI 1640 enriched with human serum was used to prepare all necessary dilutions of both virus serotypes before virus was applied. Virus preparations were tested for endotoxin as previously described [13] and found to be endotoxin free.

### Infectious Center Assay

To determine the percentage of cells infected with HRV, an infectious center assay was performed, based on a protocol published previously [30]. Cells were plated at 7.5×10^5^ cells/well in 2 ml of cell culture medium to 6-well plates. To induce THP-1 differentiation to macrophages, cells were treated for 24 hr with phorbol myristate acetate (PMA) (Sigma) at a concentration of 200 nM. Following the differentiation, the PMA-containing medium was removed and replaced with an equal volume of fresh medium, and the cells were rested for an additional 24 hours. THP-1 PMA-differentiated macrophages were infected with HRV16, HRV39, HRV1A, or HRV2 at a multiplicity of infection (MOI) of 1 or 10 in 100 µL PBS, and virus was adsorbed 1 hour at 37°C with shaking every 15 minutes. Following adsorption, cells were exposed to 0.25% Trypsin-EDTA (Gibco) and diluted to 10, 100, and 1000 cells per 100 µL. Suspended cells were adsorbed for 1 hr in duplicate on monolayers of HeLa cells (ATCC) prepared in 6-well tissue culture plates. Following this adsorption, culture medium was removed and replaced with a semisolid overlay of 1x DMEM (Gibco) with 1% penicillin/streptomycin and 1.9% Type VII Agarose (Sigma). The molten overlay was allowed to cool and plates were incubated for four days at 34°C. Following the incubation, monolayers were fixed in 10% trichloroacetic acid (Sigma) and stained with 0.1% crystal violet solution (Sigma). The number of virus-infected macrophages was quantitated by enumeration of plaques.

### SDS-PAGE and Immunoblot Analysis

Human MDMs (1×10^6^ cells/well in a 12-well tissue culture plate) were exposed to HRV1A or HRV16 at an MOI of 10. Cell lysates were collected at 15, 30, 60, 90, and 120 minutes post-inoculation and analyzed for MAPK activation using SDS-PAGE and immunoblot as previously described [13]. Protease inhibitor cocktail and immobilized glutathione agarose beads were purchased from Sigma and Thermo Fisher Scientific (Rockford, IL), respectively. Rabbit primary antibodies were used to probe for the presence of phospho-ERK5, phospho-JNK, phospho-p38, phospho-CREB, phospho-ATF-2, phospho-C/EBP-alpha (Cell Signaling Technology, Danvers, MA) and GRB2 (Santa Cruz Biotechnology, Santa Cruz, CA) as a protein loading control. Blots were visualized using horseradish peroxidase-coupled goat anti-rabbit secondary antibodies (Santa Cruz Biotechnology) and Supersignal™ chemiluminescence substrate reagents (Thermo Fisher Scientific) on a KODAK Image Station 4000 MM (Kodak, Rochester, NY) and Kodak MI Imaging Software (version 4.0.3).

### Enzyme-linked Immunosorbant Assay

HRV16 and HRV1A were applied at a MOI of 10. Following a 24-hour incubation, the supernatants were removed to cluster tubes and stored at −20°C until sandwich enzyme-linked immunosorbant assay (ELISA) could be performed to probe for CCL2, CCL20, CXCL10, and IL-10 release. Anti-CCL2, anti-CCL20, anti-IL-10, and anti-CXCL10 antibody pairs and purified protein standards were acquired from R&D Systems (Minneapolis, MN). Half-size 96 well enzyme immunoassay (EIA) plates were coated overnight at 4°C with coating buffer containing the concentrations of monoclonal capture antibody recommended by the manufacturer. The plates were washed three times with 1x phosphate buffered saline with Tween 20 (PBS-T) to remove excess antibody, and a standard curve of successive 1∶2 dilutions of protein was prepared. Standards and experimental samples were added to the 96-well plate in triplicate at 25 µL per well, and the plate was incubated at 4°C overnight. Plates were wasted three times in 1x PBS-T and monoclonal detection antibody was added to the plate per manufacturer recommendations and incubated at room temperature for 1 hour. The plate was again washed three times in 1x PBS-T. A 1∶10,000 dilution of streptavidin-HRP was added to the wells, and the plate was incubated for 20 minutes. The plate was then washed three times in 1x PBS-T, and 50 µL of TMB component HRP microwell substrate solution (BioFX, Owing Mills, MD) was added to each well. When a blue color developed such that a gradation between standards could be visually detected, the reaction was stopped with 1 M hydrochloric acid. Optical density (absorbance) was read at 450 nm using a Triad microplate reader. Protein concentrations were calculated by averaging the triplicate values and interpolating from the standard curve.

### Flow Cytometry Measurements of Mitochondrial Membrane Potential

Measuring mitochondrial membrane potential with the fluorophore rhodamine 123 in combination with flow cytometry is a widely accepted way to characterize mitochondrial function [36]. Primary human MDMs were treated with HRV16 or HRV1A at an MOI of 10 for one or eight hours. Cells were disassociated by trypsin and centrifuged for two minutes at 500×G. The supernatant was removed, and the pellet resuspended in medium at approximately 1×10^6^ cells/ml by gentle agitation and kept on ice until the experiment was performed. Cells were transferred to flow cytometry tubes (500,000 cells/tube) and rhodamine 123 (Invitrogen) was added to a final concentration of 50 µM to assay mitochondrial membrane potential. Flow cytometry was performed by measuring 10,000 cells on a BD Biosciences FACSCaliber flow cytometer. Histogram statistics were analyzed by the program CellQuest (Becton Dickinson).

### RNA Sequencing and Analysis

Human MDMs were infected with HRV1A, HRV16, or mock at an MOI of 10 for eight hours, washed 5x in PBS, and RNA was harvested by extraction with Trizol (VWR Scientific, Radnor, PA) and purified by RNeasy column elution per manufacturer protocol, including the optional DNase I digestion (Qiagen, Valencia, CA). RNA quality was ensured by Agilent Bioanalyzer 2100 analysis, and RNA samples were submitted to the Columbia Genome Center for stranded ribominus library preparation and 30 million 100-base single-end reads on the Illumina HiSeq platform. Sequencing reads were mapped against the reference genome (hg19 assembly) using the BWA [37]. Only uniquely placed reads were used for further analysis. Cisgenome v2.0 was used to calculate reads per 1000 base pairs of transcript per million reads sequenced (RPKM) values for all RefSeq annotated transcripts [38]. To avoid transcripts with zero mapped tags to interfere with logarithmic transformation of read counts, 0.1 read was added to each transcript. Raw read counts were normalized to the transcript length and sequencing depth and quantile normalized. RNA-sequencing data were deposited to the NCBI Sequence Read Archive database under accession number GSE55271.

### RNA Extraction and Quantitative RT-PCR

RNA was extracted from cells using the RNeasy method, and cDNA synthesized using the Omniscript Reverse Transcriptase Kit (Qiagen) and oligo(dT)15 primer (IDT, Coralville, IA) according to the manufacturer’s instructions. Real-time quantitative PCR (qPCR) was performed using an ABI 7500 (Applied Biosystems, Foster City, CA) using SYBR Green Universal PCR Master Mix and No AmpErase UNG (Applied Biosystems). Primers for CCL2, CCL20, CXCL10, IL-10 and β-actin were purchased from Qiagen. The thermal cycler was set to perform an initial set-up (95°, 10 min) and 40 cycles of denaturation (95°, 15 sec) followed by annealing/extension (60°, 1 min). After determining that all primer pairs used amplified with approximately equal efficiency (data not shown), the relative amount of mRNA for the genes of interest was determined by subtracting the threshold cycle (Ct) values from the Ct value for the internal control gene β-actin (ΔCt). Data are depicted as fold difference from untreated control using the 2^−ΔΔCt^ method.

### Statistical Analysis

ELISA protein concentrations were calculated by averaging the triplicate values for each experiment and interpolating from the standard curve, with differences between control and treatment groups determined by paired Student’s t-test for means. All statistical analyses were performed in SPSS (originally the Statistical Package for the Social Sciences) (version 16.0) using a significance cutoff of p<0.05.

## Results

### HRV16 and HRV1A Differentially Alter Gene Expression in Human Macrophages

**Figure 1:**
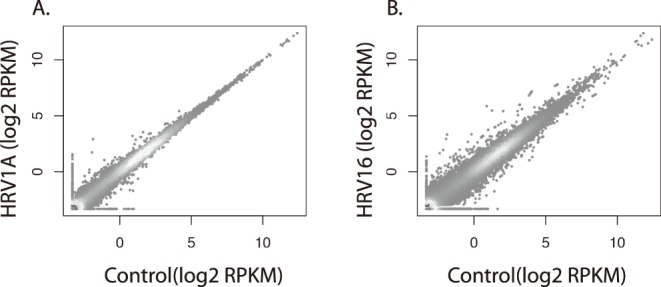
Major- and minor-group HRV produce distinct gene expression profiles in primary macrophages. Human peripheral blood mononuclear cell-derived macrophages were exposed to HRV1A, HRV16, or mock at an MOI of 10 for 8 hours. Total RNA was extracted and ribosomal RNA was depleted and sequenced on the Illumina HiSeq platform. Raw read counts for were normalized to the transcript length and sequencing depth and quantile normalized. Gene expression profiles for macrophages exposed to A) HRV1A or B) HRV16 are presented as quantile normalized read counts per transcript per kb of transcript per million sequencing tags (RPKM), log_2_ scale.

Although HRV16 and HRV1A are phylogenetically closely related, with both belonging to clade-A, and sharing 85% amino acid identity [4], [5], [39], they bind different receptors, and we have previously demonstrated that these viruses induced different biological outcomes in human primary macrophages [40]. To follow up upon this observation, we used high-throughput sequencing to identify differentially expressed genes. Human primary macrophages derived from blood were exposed to either HRV16 or HRV1A at an MOI of 10. Eight hours post-infection, total RNA was isolated and sequenced on the Illumina HiSeq platform. Substantially more genes were differentially expressed in HRV16 exposed cells compared to those macrophages exposed to HRV1A (Figure 1A and 1B) and a subset of those genes was chosen for further examination (Table 1).

**Table 1:** Select gene expression from RNA sequencing data.

Gene	Treatment Fold Difference from Control	
	HRV16	HRV1A
CXCL10	5.23	4.84
CCL8	4.95	3.74
CCL20	2.94	−2.38
CCL2	1.68	−1.52
IL-10	1.14	−1.82
MT-ND1	1.33	3.25
MT-ND6	−1.02	2.87
GBP5	2.52	1.64

### HRV Exposure Alters Mitochondrial Membrane Potential

**Figure 2:**
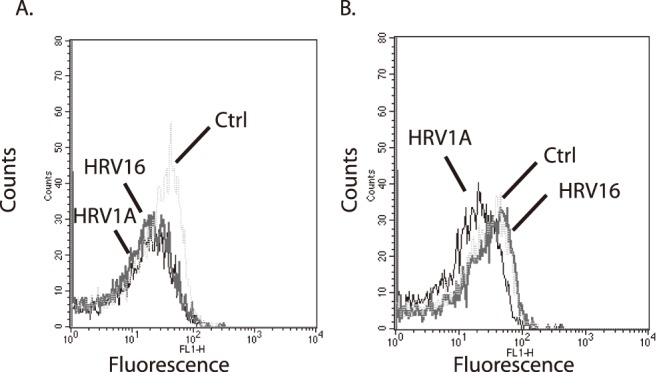
Measurement of mitochondrial membrane potential by monocyte-derived macrophages treated with human rhinovirus. Primary MDMs (1×10^6^ cells/ml) were treated with either vehicle (control, dotted line), HRV 16 (MOI of 10, grey line) or HRV 1A (MOI of 10, black line) for A) 1 hr or B) 8 hrs. Rhodamine 123 was added to the cells at a final concentration of 50 µM. The fluorescence of 10,000 cells was monitored by flow cytometry. Data are representative of three independent experiments.

The initial step of a virus binding its cognate receptor can function in a similar manner as a ligand binding its receptor and subsequently activating a signal transduction pathway. Few studies have examined if the initial signal transduction induced by a virus is important to pathogenesis. HRV provides a unique opportunity to examine this possibility as different serotypes can bind at least two different receptors. Interestingly, mitochondrial genes MT-ND1 and MT-ND6 were differentially expressed between macrophages exposed to HRV16 and HRV1A (Table 1). Macrophages exposed to HRV16 and HRV1A showed more mitochondrial activity (less fluorescence) by rhodamine 123 staining than control at 1 hour post-infection (Figure 2A). Subsequently at 8 hours post-infection, HRV16 and control mitochondrial membrane potential were indistinguishable; however, the membrane potential of macrophages exposed to HRV1A remained high in accordance with the differential gene expression of mitochondrial genes observed from RNA sequencing (Table 1).

### Altered Expression of Inflammatory Cytokines Resulting from HRV Exposure

**Figure 3:**
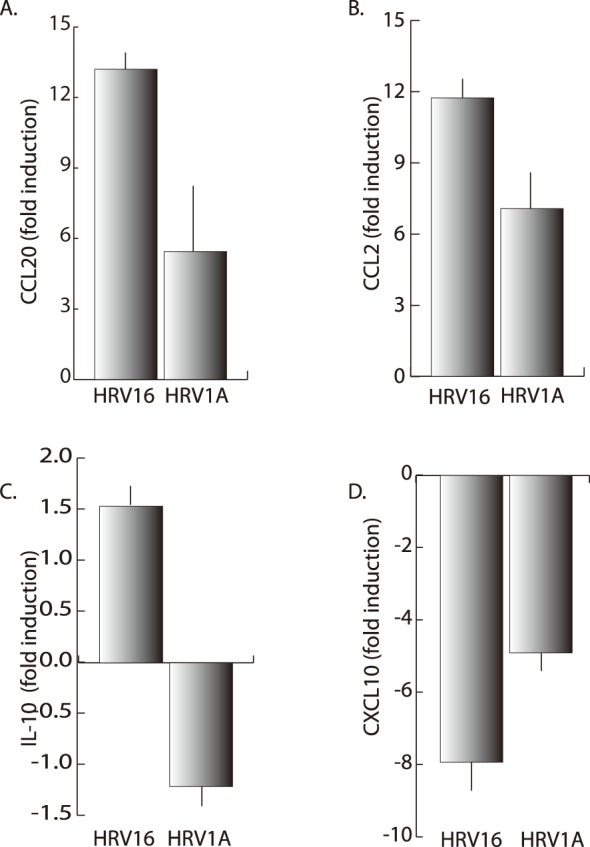
Cytokine mRNA expression by macrophages following 24 hours of exposure to HRV1A or HRV16. Primary MDMs (1×10^6^ cells/ml) were treated with HRV16 or HRV1A at an MOI of 10. Expression of A) CCL20, B) CCL2, C) IL-10 and D) CXCL10 were assayed in blood monocyte-derived macrophages by qPCR. The data are normalized to expression of the housekeeping gene β-actin and are expressed as gene expression fold change from untreated control. Error bars represent the standard error from five independent experiments.

**Figure 4:**
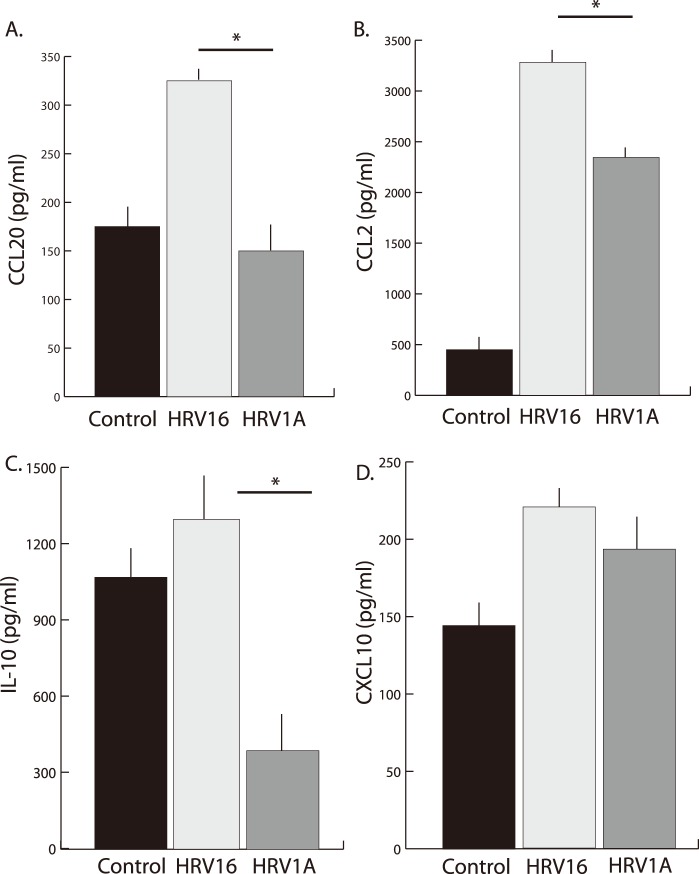
Release of inflammatory cytokines by macrophages following 24 hours of exposure to HRV1A or HRV16. Primary MDMs (1×10^6^ cells/ml) from 34 healthy donors were treated with HRV16 or HRV1A at an MOI of 10. After 24 hours, cell supernatants were analyzed for A) CCL20, B) CCL2, C) IL-10, and D) CXCL10 protein by sandwich ELISA. Data from 50 donor cell populations were pooled and analyzed by Student’s t-tests for paired samples. Significant differences (p<0.05) are indicated by*.

A variety of cytokines are produced in macrophages and epithelial cells following HRV exposure [11], [13], [28], [41]–[48]. HRV16 and HRV1A treated macrophages were analyzed by RNA sequencing which detected differential expression of several cytokines important in the HRV and asthmatic response, namely CCL20, CCL2, IL-10 and CXCL10. Differential cytokine expression and production was confirmed via quantification with both qPCR (Figure 3) and sandwich ELISA (Figure 4). As would be expected using human primary immune cells, there was a substantial range in responses across subjects.

CCL2 and CCL20 are prominent inflammatory cytokines that have been identified previously as important immune response molecules during HRV exposure in both epithelial cells [49] and macrophages [13], [40]. In particular both of these cytokines are acutely chemotactic, recruiting lymphocytes, neutrophils, and monocytes to the site of their release. Both CCL2 and CCL20 were significantly elevated with exposure to the major-group HRV16 compared to minor-group HRV1A, demonstrated both by a difference in mRNA (Figure 3A, 3B) and protein expression (Figure 4A, 4B).

IL-10 is well known for its anti-inflammatory effects [50] and may provide a replicative advantage to several viruses [51]–[54]. Interestingly, HRV1A suppressed IL-10 mRNA transcription (Figure 3C) and protein expression (Figure 4C) whereas HRV16 increased expression compared to control (Figure 4C).

Not all inflammatory mediators were expressed differentially after macrophages were exposed to the two serotypes of HRV. CXCL10 is another pro-inflammatory chemokine that is released as a result of interferon gamma (IFN-γ) production and is responsible for monocyte and macrophage recruitment as well as some anti-cancer activities [33], [46], [55]. We observed no significant differences observed in the expression of this chemokine in mRNA (Figure 3D) or proteins (Figure 4D).

### Differential MAPK Phosphorylation Induced by HRV16 and HRV1A

**Figure 5:**
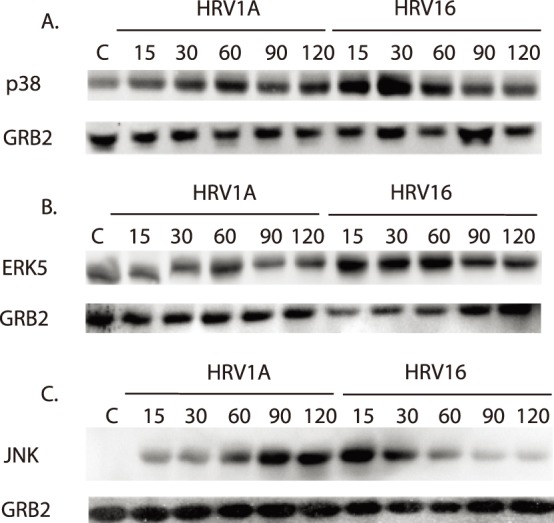
Phosphorylation kinetics of MAPKs after rhinovirus exposure. Primary MDMs (1×10^6^ cells/ml) were treated with HRV16 or HRV1A at an MOI of 10. Activation of A) p38, B) ERK5 and C) JNK were assayed by SDS-PAGE and immunoblot using anti-phospho antibodies. Equal protein loading was ensured by using total GRB2 as an internal control probing with the appropriate antibody. Each blot is representative of five independent experiments.

The MAPK p38 becomes activated as a result of stress from the environment, such as ultraviolet radiation, heat shock, or cytokines, and it is involved in promoting the production of inflammatory cytokines [13]. HRV1A elicited an increase in phospho-p38 activation within 15 minutes and continued to increase gradually up to 60 minutes. HRV16 induced a high amount of activation within 15 minutes that increased to a peak at 30 minutes. Activation was progressively decreased at the 60- and 90- minute time points. HRV1A caused a gradual increase in p38 phosphorylation whereas HRV16 induced earlier phosphorylation that peaked at 30 minutes (Figure 5A).

ERK5 activation has rarely been associated with either viruses or immune activation. However, a similar trend to that of p38 phosphorylation was observed with the activation kinetics of ERK5 following HRV exposure. When MDMs were exposed to HRV16 for a two-hour time course, there was much more initial phosphorylation of ERK5, particularly at the 15- and 30-minute time points, as compared with those exposed to HRV1A (Figure 5B).

The stress activated protein kinase (SAPK) c-Jun N-terminal kinase (JNK) is another protein kinase that is activated in response to environmental stress. In addition to its contribution to cellular differentiation and apoptosis, activated JNK is involved in inducing the production of inflammatory cytokines [20], [21], [56]. JNK phosphorylation also showed different kinetics between HRV16 and HRV1A. HRV1A induced JNK activation within 15 minutes. Activation was higher than the control at 15 minutes and then increased with each of the longer treatments. HRV16 also showed activation within 15 minutes. However, phosphorylation of JNK was highest at the 15-minute time point and decreased through the time course, indicating divergent regulation of JNK activation between virus serotypes (Figure 5C).

### HRV16 and HRV1A Induce Differential Transcription Factor Phosphorylation

**Figure 6:**
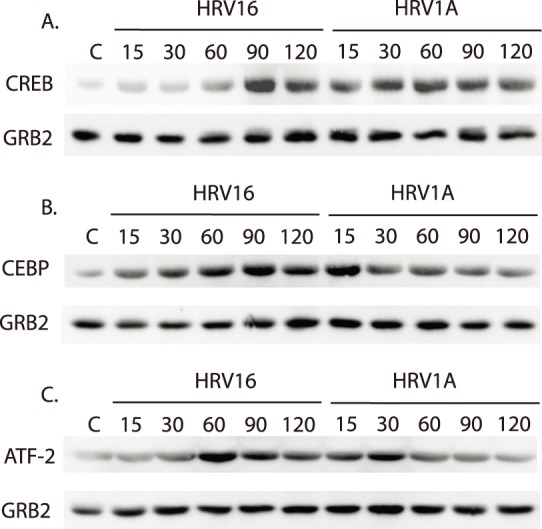
Phosphorylation kinetics of transcription factors after rhinovirus exposure. Primary MDMs (1×10^6^ cells/ml) were treated with HRV16 or HRV1A at an MOI of 10. Activation of A) CREB, B) CEBP-alpha and C) ATF-2 were assayed by SDS-PAGE and immunoblot using anti-phospho antibodies. Equal protein loading was ensured by using total GRB2 as an internal control probing with the appropriate antibody. Each blot is representative of five independent experiments.

Multiple transcription factors are phosphorylated by MAPKs, and their activation is known to be important in the general inflammatory response. Cyclic AMP is an important mediator of the inflammatory response as it is, in part, responsible for activating CREB [57]. HRV16 induced activation was low up to 90 minutes at which point phosphorylation peaked. HRV1A elicited an increase in activation within 15 minutes and a constant level of phosphorylation was maintained through 120 minutes (Figure 6A).

The transcription factor CEBP-alpha is an important mediator of inflammation and is activated through phosphorylation by a wide variety of kinases including cyclic AMP dependent kinase, the MAPKs, and protein kinase C (PKC) [58]–[61]. HRV16 induced gradual increase in activation of CEPB-alpha through 120 minutes. HRV1A, however, elicited an increase in activation of this transcription factor within 15 minutes followed by a steady decrease in the amount of phosphorylation to 120 minutes (Figure 6B).

The transcription factor ATF-2 is linked to the expression of the inflammatory cytokine CCL2 [56], [62] and also to HRV16 exposure [13], but no studies have examined the effects of HRV1A exposure on ATF-2. HRV16 elicited peak activation at 60 minutes followed by a gradual decline, whereas the activation induced by HRV1A peaked earlier than HRV16 (30 minutes) but was followed by a similar decline in phosphorylation to 120 minutes (Figure 6C).

### Major and Minor Group Rhinovirus Replicate to Different Degrees in Monocytic Cells Lines

**Figure 7:**
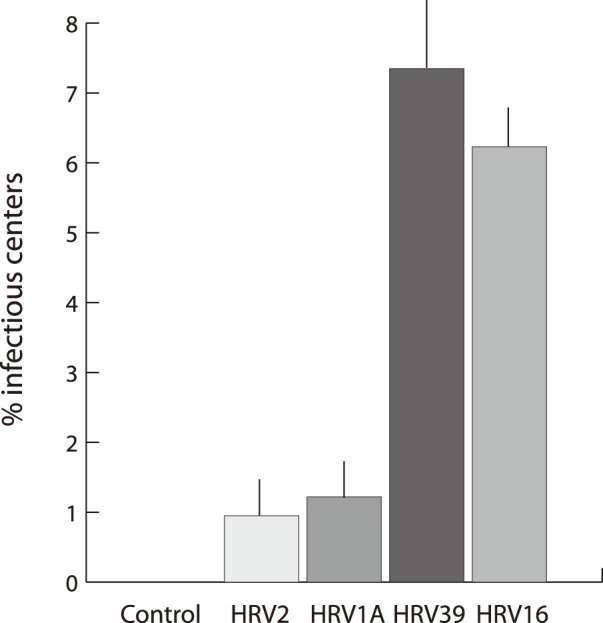
Infectious center assay in THP-1 cells. THP-1-derived macrophages were infected with the indicated HRV strains at an MOI of 10 and an infectious center assay was performed. The data are expressed as the mean percentage of infectious, virus-containing macrophages and error bars indicate the standard error of four replicates.

Laza-Stanca *et al.* noted that HRV16 replicated in the human monocytic line THP-1 with limited success but did not investigate HRV1A [29], [30]. In order to investigate replicative fecundity between major- and minor- group HRVs, we used two minor group (HRV1A and HRV2) and two major group (HRV16 and HRV39) viruses to infect PMA-differentiated THP-1 cells at an MOI of 10 for 24 hours. The results of infectious center assays of the infected THP-1 cells on HeLa monolayers demonstrated a difference in replicative capacity between the major- and minor- group rhinoviruses (Figure 7).

## Discussion

Epithelial cells are the most studied cells involved in release of HRV-induced cytokines; however, a number of studies indicate that the macrophage is also important in HRV pathogenesis [29], [31], [33], [34], [63]–[65]. Because macrophages are the second-largest cell population in the lungs (to epithelial cells) and the largest population of immune cells, they may play an important role in the inflammatory response resulting from HRV infection [13].

Several reports have demonstrated that peripheral blood monocyte-derived macrophages and alveolar macrophages behave identically to HRV exposure in signaling and cytokine secretion [13], [33], [47], [66]–[68]. This observation, coupled with both the comparative ease in harvesting MDMs and the lack of viral replication, makes this an idea cell type in which to examine the signaling processes associated with the exposure of major- and minor-group rhinovirus to their respective ICAM-1 or LDL receptors.

Although primary human macrophages are not productively infected with HRV [29], [30], they express the HRV receptors ICAM-1 and LDL-R and are known to release inflammatory cytokines including CCL2 [13], CCL5 [40], various interferons [11], and CXCL10 (IP-10) [28], [33] in response to major group HRV exposure. However, the role of these cells in minor-group HRV exposure is relatively unknown. Many studies of viral pathogenesis focus on the influence of viral nucleic acids and proteins within the host cell. Although HRV can use at least three different cell surface receptors for attachment, the idea that these receptors may play differing roles in pathogenesis has largely been unexplored.

RNA sequencing of HRV16- or 1A- exposed macrophages demonstrated a significant difference in the gene expression induced by the two viruses, suggesting different signaling pathways are activated. These differences extend to mitochondrial gene expression and mitochondrial membrane potential (Figure 2). Interestingly, mitochondrial membrane potential has been linked to anti-viral gene expression through the mitochondrial anti-viral signaling protein (MAVS) [69]–[72], providing one potential contributing mechanism for the differential gene expression between the two viruses and warrants further detailed examination.

A second possibility for differential gene expression is through the signaling initiated by major- or minor-group HRV receptor binding. We examined the MAPKs suspected to be important in inflammatory responses (p38, JNK and ERK5) after HRV16 or HRV1A exposure [20]. In all three cases, not only were the kinases phosphorylated after 15 minutes of HRV exposure, but the phosphorylation kinetics were different between major- and minor- group virus (Figure 5). This differential phosphorylation also was apparent in downstream transcription factor targets ATF-2, CREB and C/EBP-alpha (Figure 6). Differential signaling has been reported between closely related virus species that have different receptor proclivities has not been previously reported.

The work of Laza-Stanca *et al.* demonstrated that HRV16 replicates in the leukemic cell line THP-1 but not in primary macrophages [30]; however, this work did not explore minor-group viruses. We found a clear difference in viral replication between major- and minor- group rhinovirus in THP-1 cells: major-group viruses replicate with limited success whereas minor-group viruses are fully defective for replication in THP-1 cells (Figure 7). This experiment confirmed the observations in human primary macrophages and provides a tractable, homogeneous model for further examination of minor-group HRV receptor-mediated signaling as well as differences between the behavior of major- and minor- group rhinoviruses post-uncoating.

Furthermore, the major group serotypes HRV16 and HRV39 perform similarly in the THP-1 infectious center assay, as do the minor-group serotypes HRV1A and 2 (Figure 7). Taken together, these results suggest that HRV16 and HRV1A are representative of major-group and minor-group viruses, respectively, particularly within the context of macrophage signal transduction and viral replication. However, it would not be appropriate to generalize the results reported here to all major- and minor-group viruses without performing additional confirmatory studies.

Our results suggest that there is not a general viral response to HRV but rather that the macrophage responds with a virus-specific signaling response after receptor ligation. It was previously unclear if these differences translated into the development of different inflammatory microenvironments created by the viruses. Our cytokine ELISA data were quite variable, likely because any given population of primary human monocytic cells will be reacting to different immune stimuli. Indeed, Rajan et al. also noted differences in primary human monocytic cells isolated from different subjects [15]. However, a large dataset, focused on a healthy cohort, allowed us to identify statistically significant differences in the expression of CCL20, CCL2 and IL-10, all of which are important during rhinovirus infection and virally induced asthma exacerbations, after exposure with HRV16 and HRV1A. These differences did not extend to the production of CXCL10, which is also known to be involved in immune cell recruitment to sites of infection. Importantly, results obtained via qPCR did not always mirror the trends in expression observed by ELISA. However, as ELISA measures accumulation of protein, whereas qPCR measures expression at discrete time points, it is possible that differential RNA expression at time points that were not directly observed led to cytokine accumulation.

The recently discovered HRV-C clade [73], [74] is often associated with severe symptoms and asthma attacks [17]. Although the receptor for HRV-C is as of yet unidentified, the results of this study and our previous study on the Rac/TLR3/IFN axis [40] suggest several testable hypotheses. The binding of HRV-C to its receptor will trigger activation of signaling pathways described in our studies. The activation of those signaling pathways will in part lead to an altered inflammatory microenvironment. Finally, human monocytic cells will have the receptor on their surface necessary for HRV-C entry. Thus, all clades of rhinovirus will have selected receptors for entry that also trigger certain signaling pathways. This would suggest that monocytic cells, despite being non-permissive to HRV infection play an important role in HRV pathogenesis.

With these results, we propose a model wherein three separate factors affect the microinflammatory environment stimulated by HRV with respect to primary human macrophages. First, freshly activated macrophages are not always in a similar state of activation. Each individual is constantly dealing with different immunological changes resulting in isolated macrophages that respond differently to HRV challenge. Thus, a large data set was needed to observe clear differences between HRV treatments at the inflammatory mediator expression level. Second, we cannot rule out that viral capsid amino acid differences affect the production of inflammatory mediators. Finally, receptor engagement at the beginning of the viral lifecycle is important for the success of HRV infection.

In this study, macrophages have been shown to be involved in the inflammatory response related to rhinovirus infection. Specifically, HRV16 and HRV1A, which have been previously shown to be quite closely related, sharing ∼85% amino acid identity, [4], [5], [39] were shown to induce differential activation of signaling molecules in both the MAPKs and their cognate transcription factor targets, and this differential signaling resulted in differential amounts of pro- and anti-inflammatory cytokine production. Further characterization of the involved pathways and cytokine production will add to the understanding of the effects of viral infection on the host cell, add to the understanding of the asthmatic response, and offer the framework for novel treatments.

Notably, few studies have compared HRV-mediated disease severity between major- and minor-groups. However, differences have been shown between HRV groups A, B, and C in both disease prevalence [75] and type of symptoms experienced [76]. Interestingly, HRVA is responsible for the majority of cases [75] and is also the only group to contain minor-group HRVs [5]. While the higher prevalence of HRVA-mediated symptoms may be due in part to the greater number of viruses in the HRVA group, additional investigation into the differences in immune responses and disease severities between major- and minor-group HRVs is certainly warranted. The signaling differences identified in this work indicate that patients may benefit from different treatment strategies depending on the receptor binding tropism of HRV causing their infection.
